# Total Hip Arthroplasty for Low-Grade Developmental Hip Dysplasia Changes the Ipsilateral Knee Alignment on the Axial and Coronal Planes

**DOI:** 10.3390/jcm12237347

**Published:** 2023-11-27

**Authors:** Stefano Lucchini, Francesco Castagnini, Francesco Perdisa, Giuseppe Filardo, Francesco Pardo, Francesco Traina

**Affiliations:** 1 Ortopedia-Traumatologia e Chirurgia Protesica e Dei Reimpianti D’anca e di Ginocchio, IRCCS Istituto Ortopedico Rizzoli, Via Pupilli 1, 40136 Bologna, Italy; 2 Dipartimento di Scienze Biomediche e Neuromotorie, University of Bologna, 40123 Bologna, Italy; 3 Ortopedia 9, Villa Erbosa Hospital, Via dell’Arcoveggio 50, 40129 Bologna, Italy; 4 Applied and Translational Research (ATR) Center, IRCCS Istituto Ortopedico Rizzoli, Via di Barbiano 1/10, 40136 Bologna, Italy

**Keywords:** trochlea, patellar tilt, femoral anteversion, hip internal rotation, total hip arthroplasty

## Abstract

Background: There is a paucity of data regarding the post-operative influence of total hip arthroplasty (THA) on the axial and coronal alignments of the ipsilateral knee. A CT study was designed to assess the post-THA changes in axial and coronal knee alignments in low-grade dysplastic hips. Methods: Forty Crowe I–II dysplastic hips in 37 patients were assessed: a pre-operative CT scan from the fourth lumbar vertebra to the tibial plateaus was compared to a similar post-operative CT scan performed after a minimum of 2 years after THA. Results: THA implantation caused significant post-operative changes in terms of the rotation height (2 mm lowering; *p* = 0.003); center of rotation medialization (10 mm medialization; *p* < 0.001); femoral offset (11 mm increase; *p* < 0.001); femoral antetorsion (22° internal rotation; *p* < 0.001), and hip internal rotation (9° internal rotation; *p* < 0.001). The femoral axis angle deviated in the valgus (5.5° ± 1.1°, *p* < 0.001) and the mechanical lateral distal femoral angle deviated in the varus (86° ± 2.7°, *p* = 0.001). The pelvic–tibial alignment changed from 88.2° ± 11.7° to 96° ± 9.3° (*p* < 0.001). Patellar alignment was not influenced. Conclusions: In conclusion, THA imposes significant changes in low-grade dysplastic hips: all the modifications tend to neutralize the coronal alignment and, mostly, the rotational alignment, without substantial and durable variations of the patellofemoral joint. Large clinical trials should confirm whether radiological changes impact anterior knee pain and patellar stability.

## 1. Introduction

Developmental dysplasia of the hip (DDH) is associated with morphological changes in the knee joint [1,2,3,4]. The femoral condyles of dysplastic hips are smaller and asymmetric, the trochlea is more shallow, the lateral patellar shift is reduced, the patellar tilt angle is increased, and the lower limb alignment is generally in the valgus [1,2,3,4]. These morphological abnormalities tend to show a linear correlation with the degree of hip dislocation [1,2,3,4,5].

Hip joint replacement has spread worldwide and the volume of this surgical procedure is predicted to continue increasing during the next years [6,7,8]. Thus, total hip arthroplasty (THA) should consider the peculiar biomechanical features in patients affected by DDH. Nonetheless, the impact of total hip arthroplasty (THA) for DDH on the coronal and axial alignments of the ipsilateral knee is poorly ascertained. Kocabiyik et al. [9] evaluated 25 THAs with a femoral osteotomy in Crowe IV dysplastic hips. THA neutralized the coronal alignment of the knee, imposing significant changes in the femoral parameters, although with minimal hip–knee–ankle angle and mechanical axis modifications. Tokuhara et al. [10] investigated the influence of THA after DDH on the ipsilateral patellofemoral joints in 252 patients, correlating the radiological measurements to the clinical findings. Anterior knee pain occurred in 7.3% of the THAs and tended to reduce/disappear within one year. The lateral patellar tilt was significantly correlated to leg lengthening and significantly increased in painful knees. The correlation between the lateral patellar tilt and anterior knee pain was no longer observed after 3 months. Yu et al. [11] noticed in 38 dysplastic hips that femoral anteversion decreased after THA, whereas patellar tilt, lateral patellar displacement, and tibiofemoral rotation increased, even at mid-terms. Overall, these early findings suggest the importance of investigating the peculiar features of these complex patients, but the data are still lacking. 

Assessing the tridimensional hip and knee relationship through an extended CT scan should include coronal and axial alignments to detail the frontal morphotype changes as well as the main axial determinants of patellofemoral tracking. A CT study was designed to investigate post-operative changes at a minimum follow-up time of two years, in the axial and coronal knee alignments (femoral, tibial, and patellar orientations), in a cohort of THAs performed due to osteoarthritis after low-grade hip dysplasia. The hypothesis was that THA significantly changes the coronal alignment, hip internal rotation, femoral antetorsion, and lateral patellar tilt, with patellar osteoarthritis development. 

## 2. Materials and Methods

The study was approved by the IRB of the authors’ affiliated institution.

All the medical and radiological records of the THAs performed for hip dysplasia since 2004 in a tertiary center were reviewed.

The inclusion criteria were:

- THA after osteoarthritis due to low-grade developmental dysplasia of the hip (defined as a hip with a lateral center-edge angle > 20° and a Crowe classification < III) [12,13];
- Pre-operative CT scan from the fourth lumbar vertebra to the tibial metaphysis, 2 cm below the anterior tibial tubercle, in a supine position, with the hips fully extended;
- Post-operative CT scan from the fourth lumbar vertebra to the tibial metaphysis, 2 cm below the anterior tibial tubercle, performed after a minimum time span of 2 years from surgery, in a supine position, with the hips fully extended.

The two-year follow-up period was used to reduce the chronological changes in the axial knee alignment to a minimum [14].

The exclusion criteria were:

- Skeletally immature patients;
- Fixed lower limb deformities;
- Musculoskeletal abnormalities other than DDH;
- Crowe classification > II;
- Inadequate or incomplete medical and radiological assessments;
- Painful, failed, or low-performing THA;
- Post-operative radiological evaluations performed due to periprosthetic fractures or THA loosening or failure;
- Any surgical procedure around the knee;
- Advanced knee osteoarthritis (Kellgren–Lawrence [15] classification of 3 or higher; Iwano et al. [16] classification of 3).

**Figure 1:**
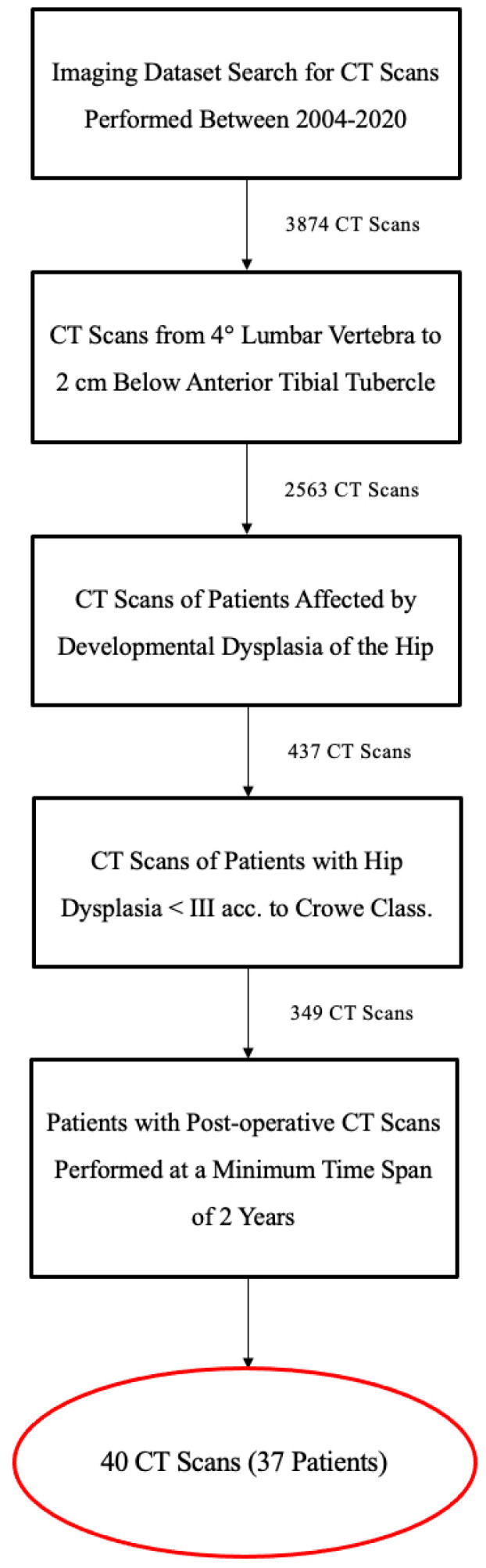
Flowchart of patient enrollments.

A consecutive retrospectively selected case series of 37 patients (40 hips) was considered (Figure 1). All the patients were then prospectively followed over time.

The demographic- and implant-related features of the cohort are specified in the table (Table 1). In all the cases, the surgical approach was an anterolateral soft tissue sparing hip approach [17] performed with the patient on a supine decubitus, a capsular suture was performed at the end of the THA implantation. In all the cases, a high hip center was adopted, aiming for a slight medialization of the center of rotation [12]. 

**Figure 2:**
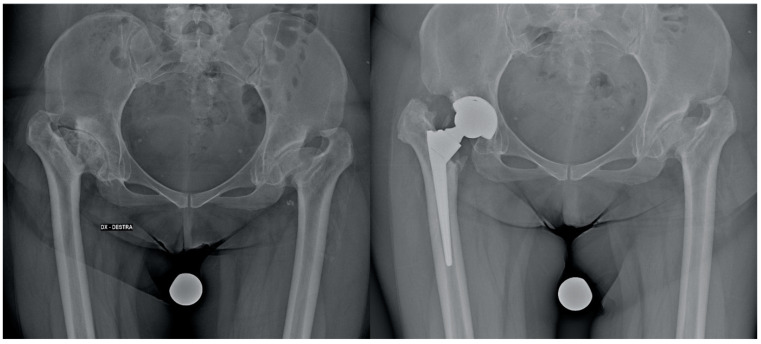
Pre-operative and post-operative X-rays of a patient treated with THA (conical stem) for hip dysplasia.

**Figure 3:**
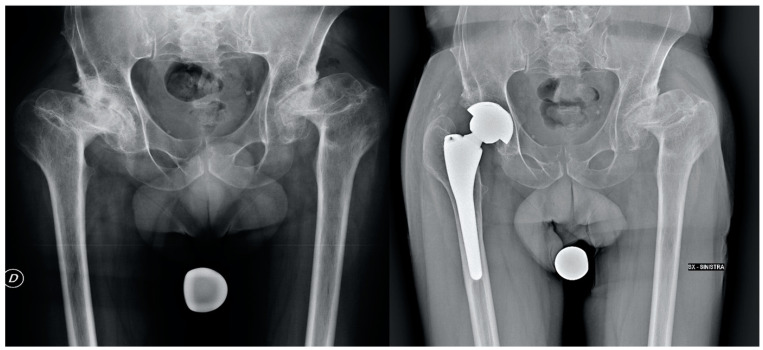
Pre-operative and post-operative X-rays of a patient treated with THA (anatomical stem) for hip dysplasia.

Stem positioning and anteversion were assessed with a 3D CT-based pre-operative planning software (HipOp) [18], with a pre-operative accurate analysis of the native femur morphology based on the CT scan (a conical stem was recommended in the small femoral canal and severe femoral anteversion [>25°] [19]) and, in the end, by the execution of intraoperative stability maneuvers, performed with femoral trial components under the direct visualization of the implant. All the implants were ceramic-on-ceramic THAs (Figure 2 and Figure 3).

Before any measurements, an intra-observer reliability test was conducted, with the observer repeating the measurements on 20 hips after 1 month. An inter-observer reliability test was also performed, involving the first 2 authors (4 and 8 years of experience, respectively). The first author was blind to the measurements of the second author.

The CT assessments were performed using Carestream Vue PACS software, version number 11.4 (Rochester, NY, USA). The pre-operative evaluation included the following steps (the measurement techniques are provided by other papers and in the figures):

- The classification of hip dysplasia according to Crowe et al. [20], the sulcus angle, the trochlear classification according to Dejour et al. [21], and the patellofemoral osteoarthritis categorized according to Iwano et al. [16] (Table 1).
- Hip biomechanics: the position of the center of rotation (CR, CR height, and CR medialization) and the femoral offset on the frontal view, and the acetabular anteversion and the femoral antetorsion (or femoral anteversion, defined as the angle composed by a line tangent to the posterior femoral condyles and the line bisecting the femoral neck) on the axial scans [22,23]. The hip internal rotation was defined as the angle between the tangent to the posterior ischial spines and the tangent to the posterior femoral condyles.
- Frontal knee alignment: mechanical and anatomic axes of the lower limb (femorotibial angles on a short-length view of the tibia), the angle between the mechanical and anatomic axes of the femur, the mechanical lateral distal femoral angle (mLDFA), the anatomic lateral distal femoral angle (aLDFA), and the Q angle [4].
- The patellar alignment: TT–TG distance, lateral patellar tilt, and patellar height according to Caton–Deschamps [1].
- The tibial rotational alignment: the knee rotation angle (KRA: the angle between the perpendicular line of the trans-epicondylar axis and the Akagi’s line) and the pelvis–tibia angle (PTA: the angle between the tangent to the posterior ischial spine and the Akagi’s line) [5,24].

The post-operative CT assessment after a minimum lifespan of 2 years was performed using a wide window width (2500–3000 HU) and a narrow window level (500–600 HU), to improve the bony visualization and reduce the metal artifacts. All the post-operative CT scans were performed for contralateral planning or noise due to ceramic-on-ceramic bearings (mean time span post-operative CT-THA: 45.6 ± 19.1 months, range: 24–70). The post-operative evaluations included hip biomechanics, frontal knee alignment, patellar alignment, and tibial rotation alignment, with the same techniques explained above.

### Statistical Analysis

The analysis was performed using SPSS 14.0 (SPSS Inc, Chicago, IL, USA). Quantitative data were reported as mean values, standard deviations, ranges of minimum and maximum, and percentiles. Qualitative data were expressed as frequencies and percentages. The reliability of the observation was assessed using the intraclass correlation coefficient (with a 95% confidence interval) with a two-way random-effect mode for consistency (inter-observer) and for absolute agreement (intra-observer). The changes between pre-operative and post-operative values were assessed using a paired *t*-test and a Wilcoxon non-parametric test (the Wilcoxon non-parametric test was used to compare the outcomes between two matched/paired groups of the population when the specific analyzed outcomes could not be considered to follow a normal distribution): the average difference, standard deviation, and 95% confidence interval were reported. The *p*-value threshold for significance was set to 0.05.

## 3. Results

### 3.1. Intra-/Inter-Observer Reliability Scores

The intra-observer reliability ranged from ICC 0.796 (95%CI: 0.359–0.945) to ICC 0.963 (95%CI: 0.863–0.991) for every measurement considered (good-to-excellent reliability). The inter-observer reliability ranged from ICC 0.752 (95%CI: 0.274–0.932) to ICC 0.956 (95%CI: 0.832–0.989) for every measurement considered (good-to-excellent reliability). Discordant cases were reviewed together and resolved by consensus.

### 3.2. Hip Biomechanics

THA implantation imposed significant post-operative changes in CR height: from 27.7 mm ± 7.8 mm (range: 16 mm–47 mm) to 25.5 mm ± 7.3 mm (range: 13 mm–41 mm) (paired *t*-test, *p* = 0.003; Wilcoxon test, *p* = 0.004) (Table 2 and Table 3). Additionally, CR medialization changed from 40.1 mm ± 6.6 mm (range: 21 mm–55 mm) to 29.9 mm ± 5.4 mm (range: 21 mm–45 mm) (paired *t*-test, *p* < 0.001; Wilcoxon test, *p* < 0.001). Femoral offset increased from 27.4 mm ± 8.5 mm (range: 9 mm–51 mm) to 37.8 mm ± 6.4 mm (range: 22 mm–50 mm) (paired *t*-test, *p* < 0.001, Wilcoxon test, *p* < 0.001). Femoral antetorsion showed greater internal rotation, from 27.6° ± 20.1° (range: −27°–78°) to 5.8° ± 4.6° (range: −21°–43°) (paired *t*-test, *p* < 0.001; Wilcoxon test, *p* < 0.001). Hip internal rotation decreased from the pre-operative value of 5.4° ± 13.2° (range: −17°–32°) to a post-operative value of −3.3° ± 9.5° (range: −23°–12°) (paired *t*-test, *p* < 0.001, Wilcoxon test, *p* < 0.001). The acetabular anteversion remained unchanged.

### 3.3. Frontal Knee Alignment

THA changed the femoral axis angle, from the pre-operative value of 4.5° ± 1.5° (range 1°–9°) to the post-operative value of 5.5° ± 1.1° (range 4°–7°) (paired *t*-test, *p* < 0.001; Wilcoxon test, *p* < 0.001) (Table 2 and Table 3). A significant post-operative change was evident for the mechanical lateral distal femoral angle, from 84.6° ± 3.2° (range: 78°–93°) to 86° ± 2.7° (range: 79°–90°) (*p* (paired *t*-test), *p* = 0.001; Wilcoxon test, *p* < 0.001). No other significant changes in the coronal alignment could be detected. 

### 3.4. Patellar Alignment

There was no significant change between the pre-operative and post-operative values regarding the lateral patellar tilt, TT–TG distance, and patellar height (Table 2 and Table 3).

### 3.5. Tibial Rotation Alignment

The KRA did not change after THA (Table 2 and Table 3). The PTA changed from 88.2° ± 11.7° (range: 61°–111°) to 96° ± 9.3° (range: 77°–115°) (paired *t*-test, *p* < 0.001; Wilcoxon test, *p* < 0.001).

## 4. Discussion

The main finding of this study was that, in low-grade dysplastic hips, THA caused coronal and axial modifications of the hip–knee alignment at a minimum follow-up time of 2 years. The patellar alignment remained substantially unchanged, whereas a femoral derotation occurred. The coronal alignment underwent modifications that did not impact the anatomical and mechanical axes. No advancement of patellar osteoarthritis occurred. 

Ollivier et al. [9,25] (in standard anatomies) and Kocabiyik et al. [9] (in osteotomized Crowe IV hips) observed some changes in the coronal hip–knee alignment after THA: the axes generally tended to neutralization, but the modifications were minimal. In the present case series, the frontal modifications were substantially close to the previous literature findings: the femoral axis increased by 1° in the valgus, the femoral offset gained 11 cm, and the mLDFA was 1.5° higher (more varus), but THA did not change the mechanical and anatomical axes. 

On the contrary, the axial alignment after THA showed more substantial variations. A 9° reduction in hip internal rotation was observed, as well as the neutralization of femoral antetorsion (a change of 22°) and a reduction in the PTA (8°). It was demonstrated that rotational malalignment may have contributed to anterior knee pain and patellar instability [26,27]. The neutralizing effect of THA on the femoral rotation (approximating the values to healthy knees) may be beneficial for dysplastic patients, improving the knee kinematics, potentially reducing anterior knee pain and stabilizing the joint. Additional studies should investigate these findings further and document the clinical relevance in terms of the changes in symptoms and functions.

Axial alignment after THA for osteoarthritis was studied by Tokuhara et al. [10] and Akiyama et al. [10,28] on 163 hips. Both the authors reported noteworthy modifications; however, unlike this case series, the internal rotation and femoral anteversion increased after THA (11° and 7°, respectively) [10,28]. The increase in the internal rotation was associated with higher femoral anteversion, leg lengthening, gender, and a postero-lateral approach) [28]. It is likely that this difference between the two case series and the present work may be ascribed mainly to the surgical approach [28]. Another striking difference between the two case series and the present work was the lateral patellar tilt: in the present report, no change was observed after THA, whereas Akiyama et al. [28] noticed an increased the lateral patellar tilt. However, Akiyama et al. [28] evaluated post-operative CT scans performed in the first six months, when the alignment changes were not definitive and the lateral patellar tilt may have showed some further adjustments [14,28]. Yu et al. [11] performed a similar study on 38 dysplastic hips, 4 years after the THA (posterolateral approach). The authors noticed a decreased femoral anteversion, as shown by the present case series, but highlighted some evident changes in the patellofemoral joint: an increase in the patellar tilt and displacement [11]. This is a contradictory outcome in comparison to this case series that can be ascribed to the change in femoral anteversion and the different tensioning of the iliotibial band [11]. 

In fact, our cases were performed using an anterolateral approach, which, unlike the posterolateral hip approach, required a lower grade of anteversion of both the stem and cup and differently influenced the soft tissues because of the distinct structures involved in the surgery. 

This study provided detailed data about the changes in axial and coronal knee alignments after THA was performed for low-grade hip dysplasia. There is a substantial lack of tridimensional assessments of the hip and knee relationship, particularly in THA after DDH: only one article investigates the tridimensional findings in a mixed population of low-grade and high-grade DDH cases. However, there are some limitations. The lack of clinical assessments does not allow us to observe if the radiological changes have some practical influence on the daily living and satisfaction levels of the patients. Moreover, the short-leg evaluation for coronal alignment was imperfect and could not properly substitute a the long-leg X-rays. The retrospective nature of the study, as well as the limited number of hips involved, were the other two notable drawbacks of the study.

## 5. Conclusions

This series provided new radiological perspectives on the tridimensional hip and knee relationship. THA resulted in some changes in the coronal, but mostly in the axial planes, in low-grade dysplastic hips. On the frontal plane, the modifications were small and tended to restore the femoral offset and neutralize the alignment. On the axial planes, the variations were much more considerable and involved hip rotation and femoral antetorsion. No other substantial and durable variations involved the patellofemoral joint. All these findings may be of clinical relevance (the possible reduction in anterior knee pain and improved patellar stability, even if not assessed in the current study) and, from the perspective of reconstructive surgery, may even modify the surgical approach to knee joints in DDH patients. Only retrospective, large clinical trials should match the radiological tridimensional changes following THA to the clinical findings.

## Figures and Tables

**Table 1:** Demographics, implants, and morphological classifications of the cohort.

Parameters	Value
Patients	37
Hips	40
Gender—women	32 (86.5%)
Weight (kg)	64 ± 8.2 (range: 58–90)
Height (cm)	159.9 ± 5.8 (range: 150–178)
BMI (kg/m^2^)	25.1 ± 2.8 (range: 19.6–30.4)
Age at THA (years)	49.8 ± 9.5 (range: 37–71)
Pre-operative Crowe classification	I: 26 (65%) II: 14 (35%)
Pre-operative Dejour classification	A: 36 (90%) B: 4 (10%)
Sulcus angle (°)	144.4 ± 10.2 (range: 118–165)
Pre-operative Iwano classification	I: 39 (97.5%) II: 1 (2.5%)
Type of stem	Anatomical (Apta Adler Ortho): 16 (40%) Conical (ADR, Smith and Nephew; Wagner Cone, Zimmer; Alata Acuta, Adler): 24 (60%)
Head size (mm)	28: 4 (10%) 32: 32 (80%) 36: 4 (10%)

**Table 2:** Pre-operative and post-operative parameters (all measurements are in mm or °). The significant changes are in bold.

Parameters	Mean	Standard Deviation	Minimum	Maximum
**Pre-Operative Hip Biomechanics**				
**CR height**	**27.7**	**7.8**	16	47
CR medialization	40.1	6.6	21	55
Femoral offset	27.4	8.5	9	51
Acetabular antiversion	20.4	8.6	4	34
Femoral antetorsion	27.6	20.1	−27	78
Hip internal rotation	5.4	13.2	−17	32
Pre-Operative Frontal Knee Alignment				
Mechanical axis	177.2	1.9	173	180
Anatomical axis	173.6	2.5	168	180
Femoral axis angle	4.5	1.5	1	9
mLDFA	84.6	3.2	78	93
aLDFA	80.2	2.8	75	87
Q angle	171.2	6.6	158	180
Pre-Operative Patellar Alignment				
TT/TG	8	3.7	3	19
Lateral patellar tilt	6.3	4.4	−2	16
Patellar height	1.1	0.1	0.9	1.3
Pre-Operative Tibial Rotational Alignment				
KRA	8.4	3.8	1	18
PTA	88.2	11.7	61	111

**Table 3:** Post-operative values are compared to pre-operative values using a Paired *t*-test and a Wilcoxon test: the significant changes are in bold (all measurements are in mm or °).

Parameters	Average Difference	Standard Deviation	*p* (Paired *t*-Test)	*p* (Wilcoxon Test)
Hip Biomechanics				
**CR height**	**2.2**	**4**	**0.003**	**0.004**
**CR medialization**	**10.2**	**6**	**<0.001**	**<0.001**
**Femoral offset**	**−10.5**	**8**	**<0.001**	**<0.001**
Acetabular anteversion	0.2	13	0.905	0.919
**Femoral antetorsion**	**21.9**	**20**	**<0.001**	**<0.001**
**Hip internal rotation**	**8.7**	**11**	**<0.001**	**<0.001**
Frontal Knee Alignment				
Mechanical axis	−0.4	2	0.205	0.290
Anatomical axis	0.6	3	0.122	0.152
**Femoral axis angle**	**−0.9**	**1**	**<0.001**	**<0.001**
**mLDFA**	**−1.4**	**3**	**0.001**	**<0.001**
aLDFA	−0.5	2	0.190	0.043
Q angle	−1.7	7	0.147	0.195
Patellar Alignment				
TT/TG	−0.6	3	0.145	0.256
Lateral patellar tilt	−1	4	0.107	0.135
Patellar height	0	0	0.819	0.859
Tibial Rotational Alignment				
KRA	0.2	4	0.739	0.416
**PTA**	**−7.8**	**10**	**<0.001**	**<0.001**

## Data Availability

The data presented in this study are available on request from the corresponding author. The data are not publicly available due to privacy.
